# Relational Agency and Ethical Professionalism Among Long-Term Care Workers: Evidence from Taiwan

**DOI:** 10.3390/healthcare14111467

**Published:** 2026-05-26

**Authors:** Mei-Lin Liao, Yi-Chun Hung, Kai-Lin Liang

**Affiliations:** 1 Taichung Veterans General Hospital (Puli Branch), Nantou County 545402, Taiwan; maylin0528@gmail.com; 2 Institute of Population Health Sciences, National Health Research Institutes, Taipei City 115603, Taiwan; sakura741106@gmail.com; 3 Department of Aging Health and Long-Term Care Management, National Chi Nan University, Nantou County 545301, Taiwan

**Keywords:** long-term care, workforce, competency, ethical professionalism, communication, Taiwan

## Abstract

**Highlights:**

**What are the main findings?**

- Relational competencies are more strongly associated with ethical professionalism than technical skills.
- Communication, psychological support, and teamwork are key predictors of ethical practice in long-term care.

**What are the implications of the main findings?**

- Workforce training should prioritize relational and psychosocial competencies alongside technical skills.
- Integrating these competencies may improve care quality and sustainability in aging societies.

**Abstract:**

**Background:** With the rapid aging of populations worldwide, strengthening the professional capacity of long-term care (LTC) workers has become a critical priority for health systems. While competency-based training frameworks are widely implemented, it remains unclear which domains of competency are most closely associated with ethical professionalism in daily care practice. **Methods**: A cross-sectional survey was conducted with 268 LTC workers across home-based, community-based, and institutional settings in Taiwan. Multiple linear regression analyses were performed to examine the associations between core competency domains and perceived ethical professionalism. **Results**: Participants reported relatively high levels of overall competency and ethical professionalism. Among the competency domains, interpersonal communication (β = 0.345, *p* < 0.001), psychological support (β = 0.184, *p* = 0.020), and teamwork (β = 0.111, *p* = 0.045) were significantly associated with ethical professionalism. In contrast, technical competencies, including physical care, daily living care, and emergency management, were not significantly associated (*p* > 0.05). **Conclusions**: The findings suggest that ethical professionalism in LTC practice is more strongly associated with relational and psychosocial competencies than with technical skills. These results highlight the importance of incorporating communication, emotional support, and teamwork training into workforce development programs. Prioritizing these competencies in training frameworks may be associated with improved care quality, workforce sustainability, and person-centered care delivery in aging societies.

## 1. Introduction

### 1.1. Global Context: Aging and the Professionalization of Long-Term Care

Population aging is one of the most significant social transformations in the Asia-Pacific region, generating growing demand for long-term care (LTC) and professional caregiving services. Strengthening the competency and professionalism of care workers has therefore become central to sustainable health and welfare systems [1]. Globally, population aging and the increasing complexity of chronic conditions have intensified pressures to ensure ethical, high-quality, and person-centered care [2]. Across OECD countries, the LTC workforce faces high turnover, heavy workloads, and emotionally demanding tasks, underscoring the need for professional development and ethical support [3]. Workforce sustainability requires policy frameworks that integrate skill development with moral responsibility [4]. Evidence from workforce development models highlights the importance of cross-sector collaboration, competency-based training, and supportive organizational structures, particularly in underserved areas [5,6].

### 1.2. Competency Frameworks and the Foundations of Professionalism

Professionalism in long-term care (LTC) is closely linked to competency frameworks that define practitioners’ essential knowledge, skills, and values [7]. These frameworks standardize expectations, guide curricula, and support professional assessment across care settings. Batt, Williams, Rich, and Tavares [7] proposed a six-step model integrating systems thinking, continuous evaluation, and the inclusion of ethical and relational competencies alongside technical performance. Their subsequent CONFERD-HP reporting guideline further emphasizes methodological transparency and stakeholder collaboration to ensure relevance in evolving healthcare contexts [8].

The effectiveness of competency frameworks depends on meaningful stakeholder engagement. Lepre et al. [9] highlighted the inclusion of practitioners, policymakers, and service users to reflect real-world values, while Al Jabri et al. [10] identified core competencies clustering around teamwork, ethics, and continuous learning. Despite the proliferation of competency frameworks, many national standards—including Taiwan’s Long-Term Care 2.0 policy—tend to prioritize measurable, technical tasks over the more nuanced, relational dimensions of care. This technical orientation creates a conceptual gap where ethical practice is often treated as a peripheral “soft skill” rather than a fundamental component of clinical excellence. There is an urgent need to examine whether high levels of technical proficiency automatically translate into ethical professionalism, or if ethical practice is rooted in distinct relational capacities.

### 1.3. Ethical Competence and Emotional Intelligence in Care Work

Ethical practice is a cornerstone of professionalism in long-term care. Care workers frequently encounter ethical dilemmas, such as balancing autonomy and safety or managing end-of-life decisions, which require both institutional and interpersonal ethical capacities [11]. McCullough’s [12] professional ethics model emphasizes moral agency and virtue-based responsibility beyond rule-based bioethics. Similarly, ethical education and reflective practice have been associated with professional accountability and patient satisfaction [13].

Professionalism in LTC also depends on soft skills and emotional intelligence. Emotional competencies—including empathy, conflict management, and emotional regulation—support ethical responsiveness and care quality [14]. Ethical competence further develops through moral sensitivity, deliberation, and action, supported by pedagogical strategies such as simulation, narrative reflection, and ethical debate [15]. Together, these studies highlight professionalism as the integration of ethical reasoning, emotion, and reflection in everyday practice.

Contemporary healthcare ethics scholarship further illuminates the moral complexity that LTC workers navigate in practice. Czekajewska et al. [16] documented how nurses and midwives encounter conscience clause dilemmas—situations where professional obligations conflict with personal moral beliefs—alongside tensions between patient autonomy and family decision-making, and moral uncertainty at the boundary between life-sustaining treatment and futile care. These findings demonstrate that ethical professionalism in healthcare is not a static competency but a dynamic, context-dependent capacity requiring continuous moral reasoning, reflexivity, and relational engagement [17]. This perspective directly supports the present study’s conceptualization of ethical professionalism as an active relational practice rather than mere procedural compliance.

### 1.4. Workplace Learning and Organizational Conditions for Professional Growth

Workplace learning theory offers an important lens for understanding the professional development of long-term care (LTC) workers. Billett [18] conceptualized learning as a social and participatory process that occurs through engagement and reflection in practice environments. Ellström, Ekholm, and Ellström [19] further distinguished between enabling and constraining learning environments, highlighting the roles of autonomy, reflection, and collaborative problem-solving in supporting adaptive learning.

Empirical studies support this perspective. Fitzgerald et al. [20] showed that team-based reflection and psychological safety are essential for effective workplace learning in LTC settings. Sarti [21] similarly emphasized job resources—such as autonomy, feedback, and supervisory support—as key drivers of engagement and retention, consistent with the job demands–resources model [3]. Recent studies further underscore the importance of relational and ethical competencies in LTC work, particularly in aging societies facing workforce instability and moral strain [3,8,22].

### 1.5. Ethical Governance and Systemic Support

Professional ethics guide individual behavior while reinforcing organizational accountability and public trust. Sfestani and Peykani [22] conceptualized professional ethics as a multi-level construct encompassing individual virtue, institutional culture, and systemic transparency. Empirical evidence from Bordbar et al. [23] supports this view, identifying management support, balanced workload, and patient-centered communication as key factors associated with adherence to ethical codes and improved care quality.

At the same time, technological and structural changes further complicate ethical practice in care settings. Vogt and König [24] noted that the use of robotic devices and information technologies in Japan’s long-term care facilities enhances efficiency but introduces new ethical challenges that require balancing technical competence with empathy. As digital transformation reshapes caregiving roles, integrating ethics, technology, and human interaction becomes central to future workforce development.

### 1.6. Relational Ethics and Interdisciplinary Professionalism in LTC

In the Asia-Pacific context, ethical care is increasingly viewed through the lens of relational ethics and community development. From this perspective, professional ethics is not merely a set of normative rules but a dynamic practice shaped by interpersonal accountability and respect for human dignity. While traditionally rooted in social work values, these principles resonate deeply with the ethic of care, emphasizing that ethical professionalism is enacted through everyday interaction and emotional attunement. By integrating relational agency into the competency framework, we can better understand how care workers navigate the moral complexities of aging societies.

### 1.7. Conceptual Integration and Analytical Framework

Drawing on competency-based education, workplace learning theory, and social work ethics, this study integrates these perspectives into a unified conceptual framework for examining ethical professionalism in long-term care work. Professional training provides the structural foundation for developing core competencies, while workplace learning environments shape how these competencies are enacted and sustained in practice. Ethical professionalism is conceptualized not as a standalone outcome, but as a relational and context-dependent dimension embedded within core competencies—particularly interpersonal communication, psychological support, and teamwork. From a social work perspective, ethical practice emerges through interaction, reflexivity, and relational accountability, providing the theoretical basis for analyzing the associations between competency domains and ethical professionalism.

### 1.8. The Taiwanese Context and Study Purpose

In Taiwan, as in other Asia-Pacific societies, long-term care is increasingly framed as part of social work practice, linking caregiving ethics with community empowerment and social development goals. The Long-Term Care 2.0 initiative has expanded community-based services but also revealed workforce challenges, including a predominance of middle-aged female workers, limited training, and high turnover, highlighting the need to strengthen both technical and ethical competencies [22].

Grounded in international frameworks, this study examines the differential associations between core competency domains and ethical professionalism among LTC workers in Taiwan. Specifically, the primary objective is to determine whether relational competency domains (interpersonal communication, psychological support, teamwork) show stronger associations with ethical professionalism than technical domains, after controlling for demographic and employment characteristics.

## 2. Methods

### 2.1. Study Design and Purpose

This study employed a quantitative, cross-sectional design to examine the relationships between professional training, core competencies, and ethical professionalism among long-term care (LTC) workers in Taiwan. The study aimed to identify factors associated with competency development and to explore how interpersonal and supportive skills relate to ethical professionalism across home-based, community-based, and institutional care settings.

A cross-sectional, self-reported approach was intentionally adopted to capture workforce-wide perceptions of competencies and ethical professionalism across diverse LTC contexts. Given the study’s focus on examining associations within an integrated competency framework—rather than establishing causal relationships—this design is appropriate for identifying patterns of alignment among competency domains at a single point in time. Self-reported measures were particularly appropriate for this study, as ethical professionalism and relational competencies involve internal moral reasoning and subjective interpersonal experiences that are often inaccessible through objective administrative data or external observation alone. By focusing on the practitioners’ own perceptions, the study captures the ‘lived’ ethical dimensions and moral agency required in daily caregiving practice. The cross-sectional design allows for the identification of associations among variables; however, causal relationships cannot be inferred. Future longitudinal studies are recommended.

### 2.2. Participants and Sampling

Participants were 268 LTC service workers recruited through purposive and snowball sampling from multiple long-term care facilities and community care stations across central and southern Taiwan. Inclusion criteria were: (1) currently employed as a certified LTC service worker or care aide, (2) having at least six months of work experience, and (3) willingness to participate voluntarily. Individuals in administrative or non-direct care roles were excluded.

Of the 268 valid responses, 88.1% were female and 11.9% male, with the majority aged between 40 and 59 years. Approximately 39.2% held a college degree or above, and more than 44% had over five years of caregiving experience. Around half (51.1%) worked in institutional care, 31.7% in home-based care, and 17.2% in community-based service settings.

### 2.3. Measures

#### 2.3.1. Core Competency Scale

The Core Competency Scale for Long-Term Care Service Workers was adapted from Taiwan’s National Core Competency Framework for Care Professionals and revised based on expert consultation. It consists of seven core competency dimensions—physical care, daily living care, emergency management, psychological support, interpersonal communication, activity facilitation, and teamwork—plus an additional domain of professional ethics.

Each item was rated on a 5-point Likert scale ranging from 1 (“strongly disagree”) to 5 (“strongly agree”), with higher scores representing greater perceived competency. Cronbach’s α coefficients for the subscales ranged from 0.87 to 0.95, and 0.96 for the overall scale, indicating high internal consistency reliability.

The Professional Ethics subscale—which served as the dependent variable in the present study—was specifically designed to capture the ethical dimensions of daily caregiving practice. Drawing on the International Federation of Social Workers (IFSW) Code of Ethics (2018) and McCullough’s [12] professional ethics model, the subscale assesses three interrelated domains: (a) respect for the autonomy and dignity of care recipients, (b) maintenance of professional boundaries and confidential practice, and (c) integration of moral responsibility into routine caregiving tasks. This operationalization reflects the consensus in the long-term care literature that ethical professionalism extends beyond rule-following and involves active moral agency enacted through everyday relational practice [11,23].

Content validity was established through review by five domain experts in long-term care and social work education. The Content Validity Index (CVI) for the professional ethics subscale exceeded 0.92. To evaluate the internal structure of the Professional Ethics subscale within the current study sample, an EFA was conducted on the subscale items using principal axis factoring with promax rotation, implemented in IBM SPSS Statistics 26.0. Items were included in reliability estimation if they loaded ≥0.40 on the extracted factor. Exploratory factor analysis confirmed that all professional ethics items loaded onto a single factor, with factor loadings ranging from 0.71 to 0.86, supporting a unidimensional structure. The Cronbach’s α for the professional ethics subscale was 0.95, indicating excellent internal consistency reliability. The subscale has demonstrated sensitivity to variation across care settings and worker experience levels in prior Taiwanese workforce studies, confirming that ethical professionalism is a variable, modifiable dimension of LTC practice rather than a fixed professional trait.

#### 2.3.2. Demographic and Work Variables

Demographic variables included gender, age, education level, marital status, and work experience (in years). Work-related variables included service setting (home-based, community-based, or institutional), employment type, and participation in professional training programs. These variables were used to explore workforce characteristics and as predictors in regression analyses.

#### 2.3.3. Instrument Validity and Ethical Considerations

The Core Competency Scale was reviewed by five domain experts in long-term care and social work education to ensure content validity and cultural relevance. The Content Validity Index (CVI) exceeded 0.90, and exploratory factor analysis confirmed the eight-dimensional structure. All procedures adhered to ethical principles aligned with the IFSW Code of Ethics (2018) and Taiwan’s Human Subjects Research Act, emphasizing respect, confidentiality, and voluntary participation. While the expert review enhanced content validity, the sample was primarily drawn from central Taiwan, and regional diversity should be considered in future replications.

### 2.4. Data Collection Procedures

Data were collected from August to October 2024 through structured self-administered questionnaires distributed by trained research assistants. Questionnaires were either paper-based or digital, depending on the preference and accessibility of participants. Participation was voluntary, and informed consent was obtained from all respondents before data collection. Respondents completed the survey anonymously within approximately 15–20 min.

### 2.5. Data Analysis

All analyses were conducted using IBM SPSS Statistics 26.0. Descriptive statistics were used to summarize demographic characteristics and competency scores. To address potential common method bias, Harman’s single-factor test was performed; no single factor accounted for more than 50% of the total variance. However, it should be noted that this test has recognized limitations as a sole indicator of common method bias [25], and the possibility of shared measurement variance cannot be fully excluded. Future research should incorporate additional methodological safeguards such as marker variables or multi-source data collection. Group differences across work settings were examined using independent-sample t-tests and one-way analysis of variance (ANOVA) with Tukey’s HSD post hoc tests. To examine relationships among workforce characteristics, core competencies, and ethical professionalism, two-stage multiple linear regression analyses were performed. Given the multiple comparisons conducted (ANOVA across eight competency domains and two regression models), readers should note that some findings may reflect Type I error inflation. Specifically, applying a Bonferroni-corrected threshold of α = 0.006 for eight simultaneous comparisons would render the teamwork association (*p* = 0.045) marginally significant; this finding should therefore be interpreted with appropriate caution and replicated in future independent samples.

To address the concern that observed competency–ethics associations might be confounded by demographic differences in the sample, an additional two-block hierarchical multiple regression analysis was conducted with professional ethics as the dependent variable. In Block 1, demographic and employment variables (gender, age, education level, work experience, and work setting) were entered as covariates. In Block 2, the seven core competency domains (excluding professional ethics) were entered simultaneously. This hierarchical approach allows estimation of the incremental variance explained by competency domains above and beyond demographic factors and provides a more rigorous test of the primary research hypotheses. Variance inflation factors (VIFs) were inspected to assess multicollinearity; all VIFs were below 5.0. A post hoc power analysis indicated that the final hierarchical regression model (R^2^ = 0.764, k = 12 predictors, N = 268) achieved statistical power exceeding 0.99 at α = 0.05, confirming adequate statistical power for detecting the observed effect sizes. The absence of a prospective a priori power calculation is acknowledged as a methodological limitation.

### 2.6. Ethical Considerations

The data used in this study were originally collected as part of routine workforce training and service evaluation activities in long-term care settings. The primary purpose of data collection (August to October 2024) was for program monitoring and workforce development rather than for research.

All participants were informed about the purpose of the data collection, and written informed consent was obtained prior to participation. The data were collected anonymously, and no personally identifiable information was recorded. Participation or withdrawal had no impact on employment or training.

Ethical approval was subsequently obtained from the Institutional Review Board of Jen-Ai Hospital, Taichung, Taiwan (Approval No. 202500023B0; approved on 19 February 2025) for the secondary analysis and academic use of the dataset. The study was conducted in accordance with the Declaration of Helsinki and Taiwan’s Ministry of Health and Welfare regulations.

## 3. Results

### 3.1. Participant Characteristics

A total of 268 long-term care (LTC) service workers participated in this study (Table 1). Most were female (88.1%), with a mean age of 44.2 years (SD = 8.7), reflecting the gendered structure of Taiwan’s LTC workforce. Nearly half had completed vocational or senior high school (45.5%), and 39.2% held a college degree or higher. Regarding experience, 44.0% had more than five years in care work. Participants were employed across institutional facilities (51.1%), home-based care (31.7%), and community stations (17.2%), indicating diverse service settings and training backgrounds representative of Taiwan’s LTC system.

### 3.2. Descriptive Analysis of Core Competencies

Table 2 summarizes descriptive statistics for eight core competency domains among LTC workers. Overall self-rated competence was moderately high (M = 4.18, SD = 0.54 on a 5-point scale). The highest scores were observed in professional ethics (M = 4.33, SD = 0.48) and interpersonal communication (M = 4.31, SD = 0.47), followed by teamwork (M = 4.27, SD = 0.50), physical care (M = 4.22, SD = 0.53), and emergency management (M = 4.17, SD = 0.58). Daily living care (M = 4.09, SD = 0.55), psychological support (M = 4.05, SD = 0.57), and activity facilitation (M = 3.98, SD = 0.61) showed comparatively lower scores, indicating potential areas for improvement. The relatively narrow SD range (0.47–0.61) suggests consistent competency levels across participants.

### 3.3. Differences in Core Competencies by Work Setting

A one-way ANOVA examined differences in competency levels by work setting (Table 3). Significant differences were found in physical care (F = 6.82, *p* = 0.001) and emergency management (F = 5.34, *p* = 0.005), with institutional care workers reporting higher scores than those in home- and community-based services. This likely reflects greater exposure to intensive care tasks and safety protocols in institutional settings. In contrast, no significant differences were observed for interpersonal communication, teamwork, or professional ethics (*p* > 0.05), suggesting that relational and ethical competencies are relatively stable across service environments. Community-based workers showed slightly higher mean scores in activity facilitation and psychological support, indicating a stronger emphasis on social engagement and emotional support in community contexts.

### 3.4. Predictors of Overall Core Competency

A multiple regression analysis examined demographic and employment factors associated with overall core competency (Table 4). The model was significant, *F*(5, 262) = 12.46, *p* < 0.001, explaining 19.2% of the variance. Education level (β = 0.215, *p* = 0.004) and work setting (β = 0.198, *p* = 0.003) were significantly associated with competency, with higher education and institutional employment linked to stronger competencies. Gender (β = 0.052, *p* = 0.192), age (β = 0.073, *p* = 0.241), and work experience (β = 0.081, *p* = 0.198) were not significant. These findings underscore the importance of educational background and institutional learning environments in competency development.

### 3.5. Predictors of Professional Ethics

To further explore the determinants of ethical professionalism, a separate regression analysis was performed using the seven core competency domains as predictors (Table 5). Consistent with the study’s conceptual positioning, this model was not intended to establish independent causal predictors of ethical practice, but to illustrate the relative structural associations between different competency domains and professional ethics within an integrated competency framework.

The overall model was statistically significant, F(7, 260) = 119.46, *p* < 0.001, explaining 76.3% of the variance in professional ethics scores (R^2^ = 0.763). Among the competency domains, interpersonal communication demonstrated the strongest association with ethical professionalism (β = 0.345, *p* < 0.001), followed by psychological support (β = 0.184, *p* = 0.020) and teamwork (β = 0.111, *p* = 0.045). These findings indicate that relational and psychosocial competencies are most closely aligned with ethical professionalism in long-term care work. Although the model explained a relatively high proportion of variance, this may reflect conceptual overlap among self-reported competency domains. Variance inflation factors (VIFs) were examined and all values were below 5, indicating that multicollinearity was not a serious concern.

In contrast, technical and task-oriented competencies—including physical care, daily living care, activity facilitation, and emergency management—were not significantly associated with professional ethics (*p* > 0.05). Rather than indicating a lack of importance, these non-significant associations suggest that ethical professionalism in care work is less closely linked to procedural proficiency and more deeply embedded in relational interaction, emotional attunement, and collaborative practice.

### 3.6. Hierarchical Regression: Competency Domains Predicting Professional Ethics After Controlling for Demographics

To evaluate whether the associations between relational competency domains and professional ethics remained robust after controlling for demographic and employment characteristics, a two-block hierarchical multiple regression was conducted (Table 6). In Block 1, demographic variables (gender, age, education, work experience, and work setting) were entered as covariates. This model was not statistically significant, F(5, 262) = 2.07, *p* = 0.072, and explained only 3.8% of the variance in professional ethics scores (R^2^ = 0.038). None of the demographic predictors reached statistical significance (all *p* > 0.05), indicating that demographic characteristics alone were not meaningful predictors of ethical professionalism in this sample.

In Block 2, the seven core competency domains were added simultaneously. The final model was highly significant, F(12, 255) = 68.41, *p* < 0.001, explaining 76.4% of the total variance (R^2^ = 0.764). The competency domains contributed a substantial incremental improvement in model fit, ΔR^2^ = 0.726, ΔF(7, 255) = 113.12, *p* < 0.001. Critically, the pattern of significant predictors was consistent with the unadjusted model: interpersonal communication (β = 0.340, *p* < 0.001), psychological support (β = 0.180, *p* = 0.023), and teamwork (β = 0.109, *p* = 0.048) remained significant predictors of professional ethics after controlling for all demographic covariates. Technical competency domains continued to show non-significant associations (all *p* > 0.05). These findings confirm that the relational competency–ethics relationship is not confounded by demographic differences in the sample.

### 3.7. Summary of Findings

Overall, the findings portray a professional workforce characterized by strong interpersonal, ethical, and teamwork competencies, supported by moderate-to-high levels of technical proficiency. Education and institutional exposure emerged as key structural factors influencing competency development, whereas relational and emotional capacities predicted ethical professionalism.

These results collectively suggest that workforce development strategies in Taiwan’s LTC sector should adopt an integrated approach—linking competency-based education, ethics-centered training, and workplace learning systems—to sustain both skill advancement and moral resilience among care workers. From a social work perspective, these findings indicate that moral competence and communication-based professionalism are essential capacities for empowering both caregivers and care recipients in community settings.

Overall, the findings indicate a workforce profile characterized by relatively strong interpersonal, ethical, and teamwork competencies alongside moderate-to-high levels of technical proficiency. Education level and institutional exposure were associated with overall competency development. Among all competency domains, interpersonal communication, psychological support, and teamwork emerged as the most theoretically salient dimensions, as they showed the strongest associations with ethical professionalism.

## 4. Discussion

### 4.1. Integrating Competency Frameworks and Ethical Professionalism

This study contributes to the growing literature suggesting that professional competency and ethical practice are not separate dimensions of long-term care (LTC) professionalism but are deeply interconnected within integrated competency frameworks. The Professional Ethics subscale used in this study—encompassing respect for dignity and autonomy, maintenance of professional boundaries, and integration of moral responsibility into daily practice—operationalizes a multidimensional construct that has been shown to vary meaningfully across care workers and contexts, and to be modifiable through targeted workforce development interventions [11,15,23]. The significant variability in professional ethics scores observed in this sample (M = 4.33, SD = 0.48) and the robust predictive relationships identified provide empirical support for treating ethical professionalism as a viable policy-relevant outcome rather than a fixed professional trait.

A key analytical contribution of this study is the hierarchical regression model, which demonstrates that the relationship between relational competencies and professional ethics cannot be attributed to demographic confounding. After controlling for gender, age, education, work experience, and care setting, the competency domains collectively explained an additional 72.6% of variance in professional ethics (ΔR^2^ = 0.726). This finding addresses the concern that observed correlations might reflect a sample-level education artifact, confirming that interpersonal communication, psychological support, and teamwork are substantively and independently associated with ethical professionalism across the workforce. The non-significance of demographic variables in Block 1 (R^2^ = 0.038, *p* = 0.072) further underscores that ethical professionalism is primarily shaped by relational practice capacities rather than structural background characteristics. Consistent with prior research, these findings highlight a distinction between task-oriented and relational competency domains [7,9], with the latter showing stronger alignment with ethical professionalism.

The strong alignment between ethical professionalism and interpersonal communication, psychological support, and teamwork resonates with educational approaches such as Entrustable Professional Activities, which conceptualize professionalism through trust, accountability, and situated practice [26]. Although the relatively high explanatory power (R^2^ = 0.763) warrants methodological caution due to shared measurement and self-report bias, it may also reflect the extent to which ethical professionalism is embedded within relational competencies. Taken together, these findings suggest that LTC workforce development may benefit from integrating competency-based education, ethics-oriented training, and workplace learning. From a social work perspective, moral competence and communication-based professionalism emerge as central capacities for empowering caregivers and care recipients in community-based care settings.

Building on these findings, the practical implications for workforce development warrant further consideration. Current competency-based training programs often emphasize technical and task-oriented skills, such as physical care and emergency response. However, the present results suggest that these domains show weaker associations with ethical professionalism in daily care practice.

Instead, relational and psychosocial competencies—particularly interpersonal communication, psychological support, and teamwork—appear to play a more central role. These competencies are essential for building trust, supporting emotional well-being, and facilitating collaborative care, all of which are critical components of person-centered care.

For policymakers and training institutions, these findings indicate the need to rebalance competency frameworks by integrating relational skill development into standard training curricula. This may include structured communication training, reflective practice sessions, and interdisciplinary teamwork exercises. Additionally, incorporating these elements into certification and continuing education programs may contribute to improved care quality and workforce retention.

Overall, strengthening relational competencies may serve as a feasible and scalable strategy to enhance both ethical practice and service outcomes in long-term care systems, particularly in rapidly aging societies.

### 4.2. Why Technical Competencies Did Not Align with Ethical Professionalism

An important finding of this study is that technical and task-oriented competencies—including physical care, daily living care, emergency management, and activity facilitation—were not significantly associated with ethical professionalism. Rather than indicating that these competencies are unimportant, this pattern suggests that ethical professionalism in long-term care is less closely tied to procedural proficiency and more deeply embedded in relational and psychosocial dimensions of care work.

Ethical challenges in long-term care practice typically arise in interpersonal contexts, such as respecting older adults’ autonomy, responding to emotional vulnerability, negotiating boundaries, and engaging in collaborative decision-making with families and care teams. These situations require moral judgment, empathy, and reflexivity rather than the execution of routinized technical tasks. From a social work perspective, this finding aligns with relational and care ethics frameworks that emphasize attentiveness, responsibility, and respect for human dignity.

Accordingly, technical competencies may be understood as necessary but not sufficient for ethical professionalism. While they ensure safety and service quality, ethical professionalism is enacted through communication, interpretation, and engagement in morally complex contexts, explaining the stronger alignment of relational and psychosocial competencies observed in this study.

### 4.3. Ethical Competence and Emotional Intelligence as Core Professional Capacities

Ethical competence emerged as a key component of professional identity and care quality. Consistent with prior research, ethical reasoning and moral sensitivity can be cultivated through educational strategies such as case-based learning, simulation, and ethical dialogue [15]. Emotional intelligence—including empathy, self-regulation, and conflict management—has also been identified as a foundational capacity shaping ethical responsiveness in care work [14]. Together, these findings highlight the importance of intentional educational and supervisory practices in supporting ethically consistent care relationships.

Empirical studies further suggest that ethical competence and moral accountability are associated with patient satisfaction, organizational trust, and reduced moral distress [26,27]. Institutional approaches that support ethical reflection and dialogue, such as the Moral Empowerment System proposed by Alonso-Prieto et al. [27], reinforce the view that ethical professionalism extends beyond procedural compliance and involves moral agency, responsibility, and integrity.

### 4.4. Workplace Learning and Organizational Support

Workplace learning was another critical factor in sustaining professionalism. This study’s findings are consistent with Billett’s [18] “learning through work” theory, which posits that engagement, reflection, and participation are essential mechanisms for developing professional competence. Ellström et al. [19] further distinguished between “enabling” and “constraining” learning environments, showing that autonomy, collaboration, and reflective dialogue promote innovation and ethical maturity. Similarly, Fitzgerald et al. [20] demonstrated that team-based reflection and psychological safety are prerequisites for effective workplace learning in LTC, transforming everyday work into opportunities for professional growth.

Sarti [21] and Hsu and Yang [28] both found that job resources such as feedback, autonomy, and supportive leadership serve as antecedents of engagement and retention among care workers. When employees experience organizational empowerment and moral recognition, they are more likely to demonstrate organizational citizenship behaviors and ethical commitment. These findings confirm the interdependence of ethical practice and workforce sustainability, supporting Llena-Nozal [3] in calling for integrated policy approaches that support improved job conditions, professional identity, and career pathways.

### 4.5. Educational Innovations and Competency-Based Training

At the educational level, several recent studies underscore the need to integrate geriatric, ethical, and emotional learning into health professional curricula. Papillon-Ferland et al. [29] revealed that geriatric competencies remain underrepresented in training programs globally, despite the growing demographic demand. Their scoping review identified a gap between competency frameworks and practice, emphasizing the importance of interprofessional collaboration and ethics-based education. Similarly, Ho et al. [30] found that students’ intention to work in aged care is significantly influenced by their exposure to experiential learning, ethical reflection, and technological competence. These findings suggest that embedding value-driven learning early in education can improve future workforce commitment and professional engagement.

Lin and Liu [31] provided additional insight by developing and validating the Eating Support for Healthcare Aides (ESHA) Questionnaire, which evaluates competencies in nutrition, feeding safety, and oral care. Their results underline that care professionalism should encompass both technical and ethical respect for residents’ autonomy and dignity in eating. These findings align with ethical frameworks that view caregiving as a moral practice rooted in respect and person-centered care [1,3].

### 4.6. Ethical Practice, Reflection, and Resilience

The interplay between ethical practice and resilience also emerged as a recurrent theme. Arjama et al. [11] and Alonso-Prieto et al. [27] both demonstrated that moral distress in LTC settings can undermine well-being and professionalism unless supported by reflective and dialogical systems. The KAP model studies by Yu et al. [32] and Hsu and Yang [28] further support this argument, showing that knowledge alone is insufficient to foster ethical practice. Instead, attitudes and self-efficacy mediate behavioral change, suggesting that emotional engagement and ethical reflection are key mechanisms linking training to ethical outcomes.

These insights also resonate with Nordic nursing scholarship, which frames caregiving as a relational and value-based profession emphasizing empathy and dignity. Embedding structured reflection sessions, storytelling, or peer discussions into ongoing LTC training could thus promote moral resilience and mitigate ethical fatigue. Additionally, developing ethical competence in clinical ethics committee settings, as demonstrated by Knox [33], further illustrates how structured training in ethical reasoning can be institutionalized to support professional growth.

Several limitations should be considered when interpreting the findings. First, the cross-sectional design precludes causal inference between professional competencies and ethical professionalism; longitudinal studies are needed to examine developmental dynamics over time. Second, reliance on self-reported measures may introduce social desirability bias and shared measurement effects. Third, although participants were drawn from diverse LTC settings, the sample was predominantly female, relatively well educated, and concentrated in central Taiwan, reflecting workforce characteristics but potentially limiting generalizability to other regions, male workers, or those with lower educational attainment. Educational background may also influence interpretations of ethical concepts and self-assessments of competence. Finally, purposive and snowball sampling may have produced clustering effects and response homogeneity due to shared organizational norms. Future research using multi-site probability sampling, longitudinal designs, and multi-source data (e.g., supervisor ratings, observations, or qualitative interviews) would provide a more comprehensive understanding of ethical professionalism in long-term care contexts.

Additionally, while the Professional Ethics subscale demonstrated strong psychometric properties (α = 0.95, CVI > 0.92, unidimensional factor structure), its concurrent validity with objective performance indicators—such as supervisory ratings, care recipient satisfaction scores, or compliance records—was not assessed in this study. Future research should examine the convergent and predictive validity of the scale against behavioral and outcome-based measures to further establish its practical significance as a workforce development tool.

## 5. Conclusions

This study provides evidence that relational competency domains—specifically interpersonal communication, psychological support, and teamwork—show significantly stronger associations with ethical professionalism than technical competency domains among LTC workers in Taiwan. Critically, this differential association pattern remained robust after controlling for demographic and employment characteristics (ΔR^2^ = 0.726), indicating that the relationship is not attributable to education or care setting differences.

These findings challenge the assumption that technical skill development is the primary lever for improving ethical practice. Rather, the evidence points to relational and psychosocial competencies as the more proximal drivers of ethical professionalism in daily caregiving. From a policy perspective, this suggests that workforce development frameworks in Taiwan and comparable aging societies should intentionally integrate relational competency training—including structured communication skills, reflective supervision, and interprofessional teamwork—alongside existing technical curricula.

Future research should extend these findings through longitudinal designs, probability-based sampling, and multi-source measurement approaches—including supervisory assessments and care recipient outcome indicators—to establish the causal mechanisms and predictive validity of relational competencies in relation to ethical professionalism. The present findings offer a testable framework for evidence-informed workforce policy in rapidly aging societies.

## Figures and Tables

**Table 1:** Demographic Characteristics of Long-Term Care Service Workers (N = 268).

Variable	Category	n	%
Gender	Female	236	88.1
	Male	32	11.9
Age group (years)	20–29	24	9.0
	30–39	56	20.9
	40–49	82	30.6
	50–59	74	27.6
	60+	32	11.9
Education	Junior high or below	41	15.3
	Senior/vocational high school	122	45.5
	College or above	105	39.2
Work experience (years)	<3	57	21.3
	3–5	92	34.3
	>5	119	44.4
Work setting	Home-based	85	31.7
	Community-based	46	17.2
	Institutional	137	51.1

Note. Data are presented as frequencies and percentages. Percentages may not total 100 due to rounding.

**Table 2:** Descriptive Statistics of Core Competency Dimensions (N = 268).

Competency Dimension	Mean (M)	SD
Physical care	4.22	0.53
Daily living care	4.09	0.55
Emergency management	4.17	0.58
Psychological support	4.05	0.57
Interpersonal communication	4.31	0.47
Activity facilitation	3.98	0.61
Teamwork	4.27	0.50
Professional ethics	4.33	0.48

Note. Each item was rated on a 5-point Likert scale ranging from 1 (“strongly disagree”) to 5 (“strongly agree”). Higher scores indicate higher self-perceived competence.

**Table 3:** Differences in Core Competency Scores by Work Setting (One-Way ANOVA).

Competency Dimension	F	*p*	Post hoc (Tukey HSD)
Physical care	6.82	0.001	Institutional > Home, Community
Daily living care	1.24	0.293	n.s.
Emergency management	5.34	0.005	Institutional > Community
Psychological support	2.11	0.123	n.s.
Interpersonal communication	1.89	0.153	n.s.
Activity facilitation	0.97	0.379	n.s.
Teamwork	1.34	0.264	n.s.
Professional ethics	2.47	0.087	n.s.

Note. n.s. = not significant at *p* < 0.05. Work setting categories include home-based (n = 85), community-based (n = 46), and institutional (n = 137) care. For physical care, group means: Home M = 4.11 (SD = 0.55), Community M = 4.09 (SD = 0.52), Institutional M = 4.32 (SD = 0.50). For emergency management: Home M = 4.02 (SD = 0.61), Community M = 4.05 (SD = 0.57), Institutional M = 4.28 (SD = 0.54).

**Table 4:** Multiple Regression Analysis of Demographic Predictors of Overall Core Competency.

Predictor	B	SE B	β	t	*p*
(Constant)	3.54	0.42	—	8.43	<0.001
Gender (female = 1)	0.08	0.06	0.052	1.31	0.192
Age	0.02	0.01	0.074	1.18	0.238
Education level	0.11	0.04	0.215	2.94	0.004
Work experience	0.03	0.02	0.066	1.12	0.263
Work setting (institutional = 1)	0.21	0.07	0.198	2.99	0.003

Model summary: F(5, 262) = 12.46, *p* < 0.001, R^2^ = 0.192, Adjusted R^2^ = 0.174. Note. Dependent variable = overall core competency score (mean of eight subscales).

**Table 5:** Multiple Regression Analysis of Core Competency Dimensions Predicting Professional Ethics.

Predictor	B	SE B	β	t	*p*
(Constant)	4.39	0.99	-	4.42	<0.001
Physical care	0.03	0.02	0.075	1.19	0.234
Daily living care	0.05	0.03	0.132	1.49	0.138
Emergency management	0.10	0.05	0.104	1.89	0.059
Interpersonal communication	0.46	0.10	0.345	4.55	<0.001
Psychological support	0.18	0.08	0.184	2.34	0.020
Teamwork	0.12	0.06	0.111	2.02	0.045
Activity facilitation	0.00	0.04	0.002	0.03	0.976

Model summary: F(7, 260) = 119.46, *p* < 0.001, R^2^ = 0.763, Adjusted R^2^ = 0.756. Note. Dependent variable = professional ethics score. Higher β values indicate stronger positive relationships between competency dimensions and ethical practice.

**Table 6:** Hierarchical Multiple Regression Analysis: Competency Domains Predicting Professional Ethics After Controlling for Demographics.

Predictor	B	SE B	β	t	*p*
Block 1: Demographic Variables					
Gender (female = 1)	0.02	0.05	0.018	0.41	0.684
Age	0.01	0.01	0.063	1.02	0.309
Education level	0.07	0.04	0.142	1.74	0.083
Work experience	0.02	0.02	0.049	0.88	0.381
Work setting (institutional = 1)	0.08	0.06	0.082	1.39	0.166
Model summary (Block 1): F(5, 262) = 2.07, *p* = 0.072, R^2^ = 0.038, Adjusted R^2^ = 0.019					
Block 2: Core Competency Domains (added to Block 1 covariates)					
Physical care	0.03	0.02	0.072	1.15	0.251
Daily living care	0.04	0.03	0.121	1.37	0.172
Emergency management	0.09	0.05	0.098	1.81	0.071
Interpersonal communication	0.45	0.10	0.340	4.48	<0.001
Psychological support	0.17	0.08	0.180	2.28	0.023
Teamwork	0.11	0.06	0.109	1.98	0.048
Activity facilitation	0.00	0.04	0.001	0.02	0.984
Model summary (Block 2): F(12, 255) = 68.41, *p* < 0.001, R^2^ = 0.764, Adjusted R^2^ = 0.750; ΔR^2^ = 0.726, ΔF(7, 255) = 113.12, *p* < 0.001					

Note. Dependent variable = professional ethics score. Block 1 includes demographic covariates only. Block 2 adds the seven core competency domains simultaneously. β = standardized regression coefficient; ΔR^2^ = incremental R^2^ contributed by Block 2. All variance inflation factors (VIFs) < 5.0.

## Data Availability

The data presented in this study are available on request from the corresponding author. The data are not publicly available due to privacy restrictions related to the anonymity of research participants.
